# Microvasculopathy in spinal muscular atrophy is driven by a reversible autonomous endothelial cell defect

**DOI:** 10.1172/JCI153430

**Published:** 2022-11-01

**Authors:** Haiyan Zhou, Ying Hong, Mariacristina Scoto, Alison Thomson, Emma Pead, Tom MacGillivray, Elena Hernandez-Gerez, Francesco Catapano, Jinhong Meng, Qiang Zhang, Gillian Hunter, Hannah K. Shorrock, Thomas K. Ng, Abedallah Hamida, Mathilde Sanson, Giovanni Baranello, Kevin Howell, Thomas H. Gillingwater, Paul Brogan, Dorothy A. Thompson, Simon H. Parson, Francesco Muntoni

**Affiliations:** 1 Genetics and Genomic Medicine Research and Teaching Department,; 2 NIHR Great Ormond Street Hospital Biomedical Research Centre,; 3 Infection, Inflammation and Rheumatology Section, and; 4 Dubowitz Neuromuscular Centre, Developmental Neurosciences Research and Teaching Department, Great Ormond Street Institute of Child Health, University College London, London, United Kingdom.; 5 Institute of Medical Sciences, University of Aberdeen, Foresterhill, Aberdeen, United Kingdom.; 6 Centre for Clinical Brain Sciences and; 7 Edinburgh Medical School, Biomedical Sciences, College of Medicine & Veterinary Medicine, University of Edinburgh, Edinburgh, United Kingdom.; 8 Microvascular Diagnostics, Institute of Immunity and Transplantation, University College London, London, United Kingdom.; 9 Tony Kriss Visual Electrophysiology Unit, Clinical and Academic Department of Ophthalmology, Sight and Sound Centre, Great Ormond Street Hospital, London, United Kingdom.

**Keywords:** Neuroscience, Neuromuscular disease

## Abstract

Spinal muscular atrophy (SMA) is a neuromuscular disorder due to degeneration of spinal cord motor neurons caused by deficiency of the ubiquitously expressed SMN protein. Here, we present a retinal vascular defect in patients, recapitulated in SMA transgenic mice, driven by failure of angiogenesis and maturation of blood vessels. Importantly, the retinal vascular phenotype was rescued by early, systemic SMN restoration therapy in SMA mice. We also demonstrate in patients an unfavorable imbalance between endothelial injury and repair, as indicated by increased circulating endothelial cell counts and decreased endothelial progenitor cell counts in blood circulation. The cellular markers of endothelial injury were associated with disease severity and improved following SMN restoration treatment in cultured endothelial cells from patients. Finally, we demonstrated autonomous defects in angiogenesis and blood vessel formation, secondary to SMN deficiency in cultured human and mouse endothelial cells, as the underlying cellular mechanism of microvascular pathology. Our cellular and vascular biomarker findings indicate microvasculopathy as a fundamental feature of SMA. Our findings provide mechanistic insights into previously described SMA microvascular complications, and highlight the functional role of SMN in the periphery, including the vascular system, where deficiency of SMN can be addressed by systemic SMN-restoring treatment.

## Introduction

Spinal muscular atrophy (SMA) is an autosomal recessive neuromuscular disorder caused, in approximately 95% of patients, by homozygous deletion of the survival motor neuron 1 gene (*SMN1*) (1). The neuropathological hallmark of SMA is the selective degeneration of lower motor neurons, with ensuing muscle atrophy and weakness. In the most common and severe form (type 1 SMA, or Werdnig-Hoffmann disease), children die within the first 2 years of life, without acquiring the ability to sit. Relatively milder forms are intermediate, or type 2, SMA, in which children sit but are unable to walk; and type 3 SMA, in which ambulation is acquired although often lost with age (2). SMA type 1 is the most common genetic cause of infant mortality, with an incidence of around 1 in 6,000 live births and a carrier frequency of about 1:35 in the White population (3).

Notable breakthroughs in therapy for SMA have recently been achieved using therapeutic interventions aimed at increasing SMN protein levels (4). Nusinersen is an antisense oligonucleotide (AON) drug that has been approved by the US Food and Drug Administration (FDA) and European Medicines Agency (EMA) for the treatment of patients with SMA (5, 6). It increases full-length SMN protein expression by targeting the intronic splicing silencer N1 element (ISS-N1) in intron 7 of the *SMN2* gene and augmenting exon 7 inclusion in the mature *SMN2* mRNA (7, 8). Nusinersen is delivered exclusively to the central nervous system (CNS) by regular intrathecal injections. *SMN1* gene replacement therapy using adeno-associated virus (AAV) is another effective therapy for SMA (9–11). Onasemnogene abeparvovec (AVXS-101), an AAV9-SMN gene therapy drug approved by the FDA and the EMA, is a one-off treatment delivered intravenously in infants with SMA type 1, and is able to cross the blood-brain barrier. The orally bioavailable small-molecule *SMN2* splice modulators risdiplam and branaplam are also effective; risdiplam has been approved by the FDA and the EMA (12, 13). In contrast to nusinersen, which acts exclusively in the CNS, both onasemnogene abeparvovec and risdiplam are delivered systemically and therefore address SMN deficiency in both the CNS and peripheral organs (14), although differences in peripheral biodistribution between these two therapies exist.

The SMN protein is ubiquitously expressed. While SMA is primarily a lower motor neuron disease, an increasing body of evidence from clinical and animal-based studies indicates a contribution of peripheral organs to the complex pathogenesis of the disease (15–17). It is therefore important to determine which organs have a clinically relevant requirement for SMN protein, as different therapies may differentially address the SMN deficiency in discrete tissues and organs.

Vascular-related defects have been reported in severe cases of infantile SMA, and in mouse models of SMN deficiency. These include digital necrosis, distal vascular thrombosis, and skin necrosis (18–20); tail and ear necrosis and decreased density of capillaries in spinal cord and intestine in SMA mice (21–23); and decreased density of capillaries in skeletal muscle from both patients and mice (22, 24). Cardiovascular abnormalities, including congenital heart disease and heart failure, have also been reported in severe SMA patients and SMA mice (19, 25–27). These defects result in widespread tissue hypoxia in animal models of SMA, to which motor neurons appear to be particularly susceptible (28). In addition, changes in the expression of some vascular-related factors have been reported in blood samples of patients with SMA, suggesting that the vascular abnormalities could be pervasive in the SMA patient population (29).

Given the essential nature of the blood supply to the spinal cord and brain, and the sensitivity of motor neurons to defects in oxygen supply from disordered capillary networks, it is important to clarify the nature of a microvascular disease phenotype in SMA. Here, we used pediatric retinal imaging to reveal defects in retinal vascularization in SMA patients. We recapitulated the phenotype in mouse models of severe SMA and demonstrated defective angiogenesis and maturation of the primary retinal vasculature. In addition, we show that antisense therapy, delivered systemically at birth to increase SMN protein expression, can normalize the microvascular defect in these mice. Using blood samples from patients, we go on to show increased vascular damage and decreased vascular repair. Importantly, this vascular damage was correlated with disease severity and *SMN2* copy number. Finally, using 2 different cellular systems, we show that the vascular defect is driven by a cell-autonomous defect in the ability of SMN-depleted endothelial cells to form vessels.

## Results

### A retinal vascular phenotype in SMA patients.

Retinal imaging is a reliable indicator of vascular health and disease in the CNS (30). Conventional color fundus photography captures a field of view of 30° to 45°, but here we used ultra-widefield imaging to capture an extended image of the retinal vasculature, with an angular field of view of up to 200°. This allowed us to assess larger areas of the retina, but as the imaging equipment requires postural cooperation from participants to position the eye at the optimal distance for focus and field of view, we restricted our study to SMA type 2 and 3 patients (31). The patients had normal visual acuity and did not complain of any visual difficulty.

**Figure 1:**
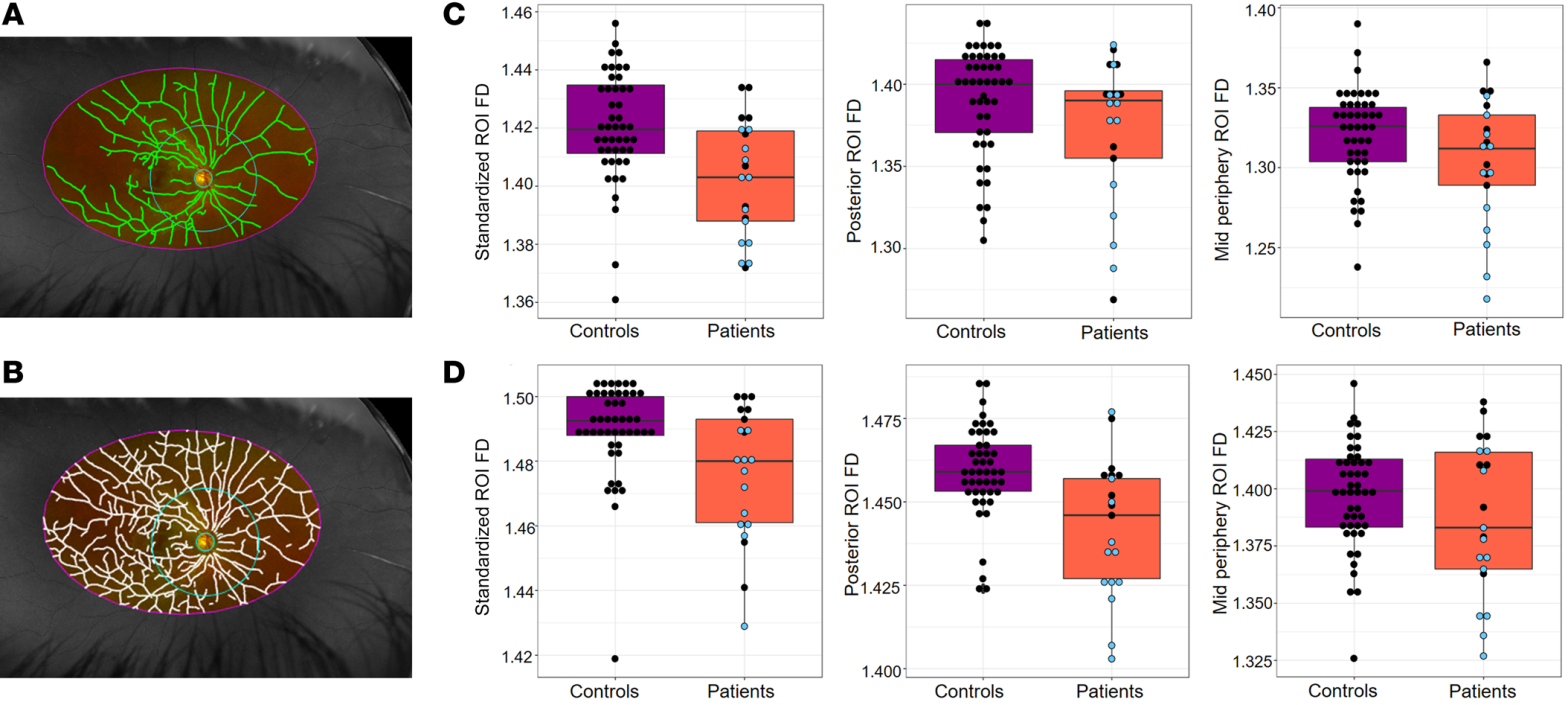
Retinal imaging and data analysis in SMA patients. The vessel skeletons produced using 2 different automatic vasculature segmentation methods are shown: (**A**) Skeleton (in green) from methods of Pellegrini et al. (34) segments only prominent vessels in the image. (**B**) Skeleton (in white) using IterNet neural network (33) segments discrete and prominent vessels. Ultra-widefield retinal images in grayscale are shown from the right eye of the same individual. (**C** and **D**) Fractal dimensions (FDs) were calculated by segmentation method of Pellegrini et al. (**C**) and IterNet neural network (**D**) from 3 regions of interest (ROIs) outlined by colored lines: standardized ROI (magenta outline), posterior ROI (cyan outline around the optic nerve head), and midperiphery ROI (between cyan and magenta). For each segmentation method, 3 box plots show the distribution of corresponding FD calculated from each ROI in SMA patients (orange boxes, *n* = 21) compared with controls (magenta boxes, *n* = 46). Blue dots, type 2 SMA patients (*n* = 12); black dots, type 3 (*n* = 9).

We analyzed ultra-widefield images from 21 eyes of 11 SMA patients (images from 1 eye were excluded owing to a poor view of the vasculature obstructed by eyelash) and 46 eyes of 23 healthy control children, to generate fractal dimension (FD) measures of retinal vessel branching complexity. FD is a unitless index of the degree of complexity and hence space filling by the vessels, on the retinal surface. FD is a well-established parameter for objectively characterizing the complexity of the retinal microvasculature (32) and is used in neurogenerative diseases as a surrogate for cerebral microvasculature changes (31). FD values generally range between 1 and 2, and we considered a change of 0.01 to be biologically relevant based on our previous experience (E. Pead, unpublished observations). A lower FD value indicates a pattern of retinal vessels that is less space filling, analogous to increased lacunarity or mesh size. We calculated the FD in 3 regions of interest (ROI): a standardized ROI for ultra-widefield images (VAMPIRE-UWF), further subdivided into a posterior ROI (centered around the optic disc) and a midperipheral ROI (the difference in area between the standardized and the posterior ROI) (see Methods). SMA patients had a significantly lower FD in the standardized ROI using 2 different methods of segmentation (β = –0.019, *P* < 0.001 by the Pellegrini method; β = –0.017, *P* = 0.001 by the IterNet method; Table 1) (33, 34), indicating that the vascular patterning was less space filling within this ROI (Figure 1). Using the IterNet method of segmentation that detects smaller branching vessels (33), SMA patients exhibited a significantly lower FD in the posterior ROI compared with controls (β = –0.018, *P* < 0.001) (Figure 1). The mean FD was lower in the midperiphery zone, but not to a statistically significant degree.

The significant difference in FD indicates a less dense and less complex primary retinal vasculature in SMA patients when compared with age-matched control children.

### Demonstration of the retinal vascular phenotype in SMA mice.

**Figure 2:**
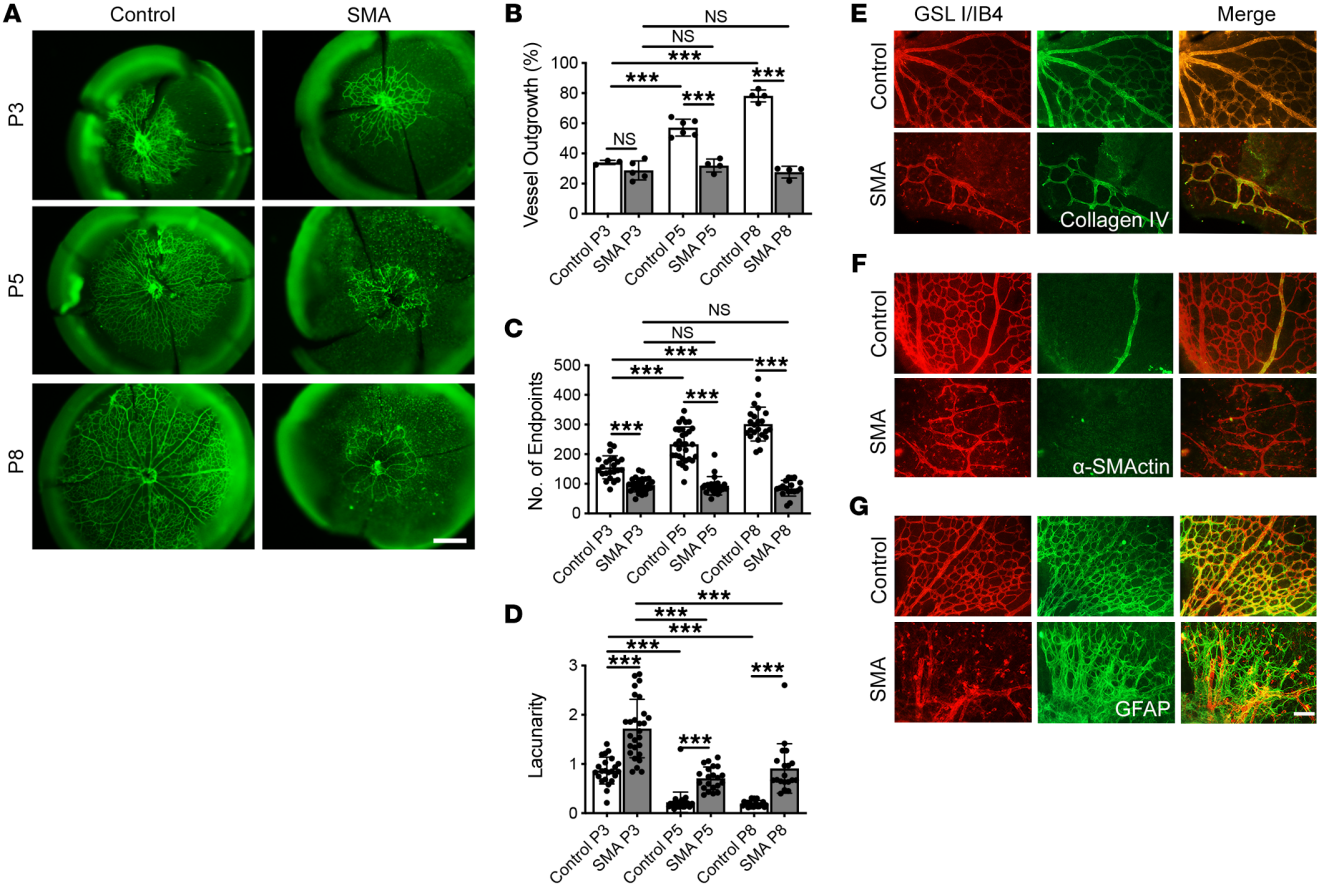
Abnormal postnatal development of retinal vasculature in a mouse model of SMA. (**A**) Retinas were collected from SMA mice and healthy littermate controls at P3, P5, and P8 and stained with GSL I/IB4 lectin (green). (**B**–**D**) Quantification of the retinal vasculature using AngioTool on vessel outgrowth (**B**), number of microvessel endpoints (**C**), and lacunarity (**D**) in SMA retinas compared with controls at P3, P5, and P8. (**E**) Costaining for vessels with GSL I/IB4 (red) and basement lamina with collagen IV (green). (**F**) Costaining for vessels with GSL I/IB4 (red) and smooth muscle with α-smooth muscle actin (α-SMActin; green). (**G**) Costaining for vessels with GSL I/IB4 (red) and astrocytes with glial fibrillary acidic protein (GFAP; green). All images were taken from retinas from mice at P8. Data represent mean ± SEM with individual data points displayed, from at least 3 mice for each group. Scale bar: 500 μm in **A**, 50 μm in **G**. One-way ANOVA with Tukey’s post hoc test was used for data analysis. ***P < 0.001.

In order to further investigate this retinal vascular phenotype, we explored the possibility that the primary retinal vasculature defect could be replicated in the “Taiwanese” mouse model of severe SMA (35). In mice, the retinal vasculature develops exclusively postnatally, and can be demonstrated by immunohistochemical staining of whole-mount retinas, using the vascular endothelial cell marker *Griffonia simplicifolia* lectin I/isolectin B4 (GSL I/IB4). Stained retinas showed that vascular defects were readily apparent in the SMA mice from a pre-symptomatic age: postnatal day 3 (P3). In the SMA retinas the centripetal pattern of angiogenic outgrowth from the optic disc toward the periphery lagged behind the retinas of unaffected control littermates (Figure 2A). AngioTool software (36), which allows for semiautomated reconstruction and quantification of vascular networks, was used to analyze the morphometric and spatial parameters of the retinal vasculature. A dramatic disease phenotype was apparent in all parameters of vascular network outgrowth complexity measured. Vessel outgrowth, a measure of overall microvascular density expressed as a percentage of total retinal area, was significantly reduced in SMA compared with control mice at early symptomatic P5 (control, 57.1% ± 2.3%; SMA, 32.0% ± 2.2%; *P* < 0.001) and late symptomatic P8 (control, 78.2% ± 2.0%; SMA, 27.6% ± 2.0%; *P* < 0.001) but not at pre-symptomatic P3 (control, 34.1% ± 0.8%; SMA, 28.8% ± 2.8%; *P* = NS) (Figure 2B) time points. Vessel outgrowth increased in control retinas between P3 and P5, and between P3 and P8 (*P* < 0.001), but not in SMA retinas over the same periods. Numbers of microvessel endpoints, a measure of the number of likely angiogenic “tips,” were significantly reduced in SMA compared with control retinas at P3 (control, 155.7 ± 8.0; SMA, 96.2 ± 4.5; *P* < 0.001), P5 (control, 233.4 ± 10.4; SMA, 93.5 ± 6.8; *P* < 0.001), and P8 (control, 301.3 ± 11.9; SMA, 84.9 ± 6.2; *P* < 0.001) (Figure 2C). Numbers of endpoints increased in control retinas between P3 and P5, and between P3 and P8 (*P* < 0.001), but not in SMA retinas over the same periods. The decreases in microvascular outgrowth and vessel endpoints in SMA retinas indicate decreased angiogenesis and were inversely correlated with a significant increase in lacunarity (a measure of network mesh size) in SMA at P3 (control, 0.87 ± 0.06; SMA, 1.72 ± 0.11; *P* < 0.001), P5 (control, 0.22 ± 0.04; SMA, 0.71 ± 0.05; *P* < 0.001), and P8 (control, 0.2 ± 0.01; SMA, 0.91 ± 0.12; *P* < 0.001) (Figure 2D). Lacunarity decreased in control and SMA retinas between P3 and P5 (*P* < 0.001), and between P5 and P8 (*P* < 0.001), indicating a developing and increased complexity of the microvascular plexus, resulting in a highly branched and ramified structure in control but not in SMA retinas. Together these data support the patient data and reveal reduced vessel complexity (FD in the patients) in the retina as a consistent and potentially important phenotype in SMA.

Preparations in which blood vessels were labeled by GSL I/IB4 also show a population of small, isolated cells. GSL I/IB4 is an established marker of activated and quiescent microglia (37). The morphology of blood vessels and microglia is sufficiently different to make their identification possible, and this showed that there was an apparent increase in the number of microglia present in SMA retinas, especially in the periphery, where no blood vessels are present (Figure 2A).

Detailed investigation of the retinal vasculature in SMA mice showed that the naked collagen IV basal lamina tubes, indicative of vessel loss, were not present in either SMA or control mice retinas; rather, vessel and basal lamina were closely correlated (Figure 2E). This suggests that there was no evidence of the vessel regression, which would be indicative of a degenerative phenotype. Further, those vessels present in SMA retinas were failing to mature into arterioles, indicated by a lack of acquisition of smooth muscle (stained with α-smooth muscle actin) into vessel walls in comparison with the mature, differentiated arterioles in control retinas (Figure 2F). Finally, the preexisting astrocytic base (stained with glial fibrillary acidic protein), over which developing retinal vessels grow, appeared similar in control and SMA mice. However, the alignment of vessels upon this framework was very weak in SMA animals (Figure 2G). These findings are consistent with an impaired angiogenic phenotype in SMA mice. Interestingly, the preexisting, embryonic, hyaloid vasculature appeared near normal in P5 SMA mice compared with control retinas (Supplemental Figure 1; supplemental material available online with this article; https://doi.org/10.1172/JCI153430DS1).

Taken together, these data reveal an important microvascular pathology of the retina in the SMA mice, likely related to SMN protein deficiency. This phenotype, present in both SMA patients and mouse models, points toward fundamental defects in angiogenesis and microvascular development in the vessels supplying the CNS in SMA.

### Systemic in vivo AON treatment restores the retinal vascular network.

**Figure 3:**
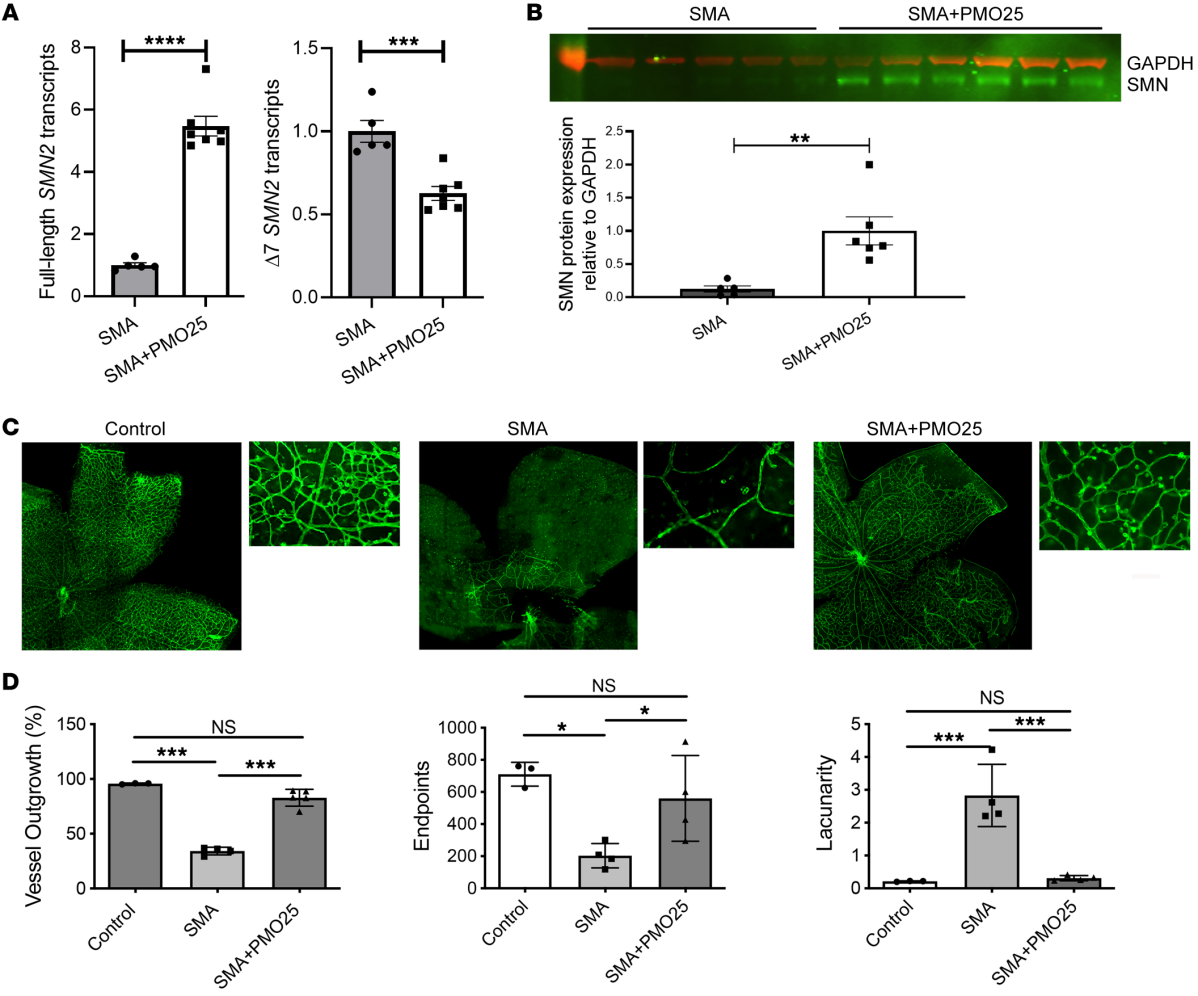
SMN restoration with antisense treatment restores retinal vasculature in SMA mice. (**A**) The full-length *SMN2* transcripts and truncated *SMN2* transcripts without exon 7 (Δ7 SMN2) were measured by quantitative real-time PCR in retinas collected from PMO25-treated SMA mice (SMA+PMO25, *n* = 7), compared with saline-treated SMA mice (*n* = 5). (**B**) Representative image of Western blotting and semiquantification of SMN protein expression in mouse retinas from SMA mice after PMO25 treatment (*n* = 6), compared with saline-treated SMA controls (*n* = 5). Mouse GAPDH protein was used as a loading control. (**C**) Mouse retinas from saline-treated SMA, PMO25-treated SMA, and healthy littermate controls were stained with GSL I/IB4 lectin (green) to indicate blood vessels of the primary vascular plexus. (**D**) The vascular plexus was quantified using AngioTool on vessel outgrowth, endpoints, and lacunarity in mouse retinas from saline-treated SMA, PMO25-treated SMA, and healthy littermate control mice. One-way ANOVA with Tukey’s post hoc test was used for data analysis. Data represent mean ± SEM, with individual data points displayed. *N* ≥ 3 eyes from at least 3 mice for each group. *P < 0.05, **P < 0.01, ***P < 0.001, ****P < 0.0001. Scale bar: 400 μm in low-power images and 200 μm in high-power inset images.

To determine whether these microvascular pathologies are amenable to therapeutic intervention, we next studied the effect of the administration of a 25-mer morpholino therapeutic AON (PMO25) on SMA mouse retinas. SMA mice were treated with a single subcutaneous injection of PMO25 at 40 μg/g at P0 as described previously (21, 38). At a late symptomatic time point of P10, retinas were collected from saline-treated SMA mice, PMO25-treated SMA mice, and healthy littermate controls. Quantitative real-time PCR on *SMN2* transcripts in mouse retinas showed a significant, 5.5-fold increase in the full-length *SMN2* transcripts (*P* < 0.0001) and a 37% decrease in the *SMN2* transcripts lacking exon 7 (Δ7 SMN2) (*P* = 0.0005) in PMO25-treated SMA mice compared with untreated SMA controls (Figure 3A), suggesting the high efficacy of PMO25 in augmenting *SMN2* exon 7 splicing. The therapeutic effect of PMO25 was further confirmed by Western blotting, with a more than 8-fold increase of SMN protein after PMO25 treatment (Figure 3B; see complete unedited blots in the supplemental material). Immunohistochemical staining of the retinal vasculature using GSL I/IB4 showed the expected, severe pathology in the SMA retinas, but also the recovery of vascularity in the PMO25-treated SMA mice (Figure 3C). This was reflected in the restoration of key parameters of network complexity at P10. Vessel outgrowth, expressed as a percentage of total area, was significantly decreased in SMA compared with healthy control mice (control, 95.7% ± 0.33%; SMA, 34.2% ± 1.7%; *P* < 0.001), and significantly restored following PMO25 treatment (SMA+PMO25, 82.9% ± 0.35%; *P* < 0.001). Numbers of endpoints were significantly decreased in SMA compared with control mice (control, 710 ± 42.9; SMA, 203 ± 37.9; *P* < 0.05) and almost completely restored after PMO25 treatment (SMA+PMO25, 559.8 ± 133.6; *P* < 0.05). Finally, the significantly increased lacunarity observed in SMA compared with control mice (control, 0.21 ± 0.01; SMA, 2.81 ± 0.47; *P* < 0.001) was also ameliorated after PMO25 treatment (SMA+PMO25, 0.31 ± 0.04; *P* < 0.001) (Figure 3, C and D).

These data support the idea that pathology of blood vessels, and constituent endothelial cells in SMA, which lie on the systemic, vascular face of the blood-brain and blood-retinal barriers, is reversible and amenable to systemically delivered SMN-restoring AON.

### Reduced retinal vascularity precedes a reduction in neuronal population and increased microgliosis in SMA mice.

**Figure 4:**
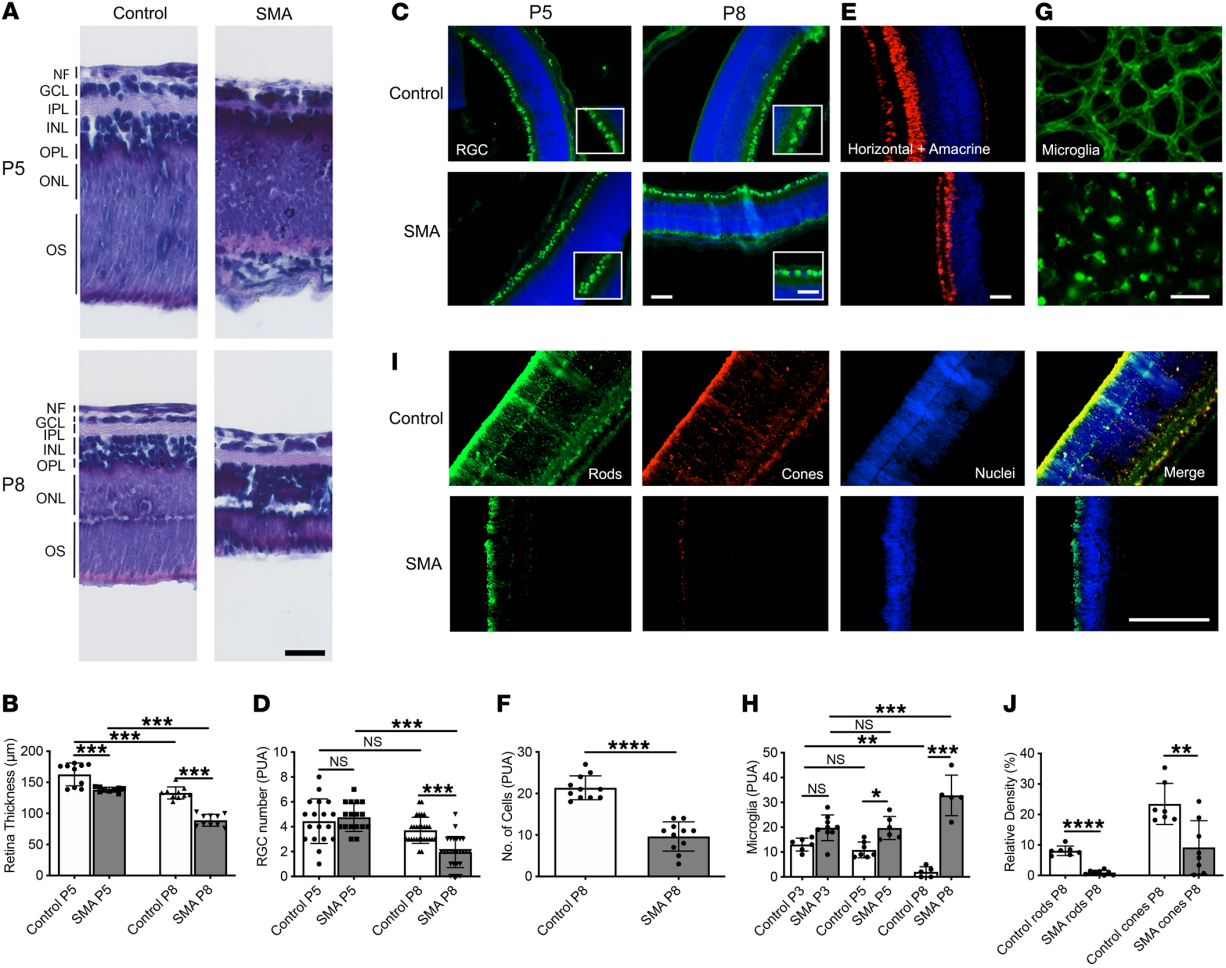
Depletion of neuronal components and increased microgliosis in SMA mouse retina. (**A**) Gross appearance of H&E-stained retina from SMA and healthy control mice at P5 and P8. GCL, ganglion cell layer; INL, inner nuclear layer; IPL, inner plexiform layer; NF, nerve fiber; ONL, outer nuclear layer; OPL, outer plexiform layer; OS, outer segment. (**B**) Quantification of retinal thickness in sections from H&E staining. (**C**) Retinal ganglion cells (RGCs) stained with BRN3a (green), with nuclei in blue. The insets show high-power fields of the RGC layer. (**D**) Quantification of RGCs per unit area (PUA) at P5 and P8. (**E**) Horizontal and amacrine cells were stained with PAX6 transcription factor (red), with nuclei in blue. (**F**) Quantification of horizontal and amacrine cells PUA at P8. (**G**) Microglia, stained with GSL I/IB4 isolectin, differentiated from blood vessels by their morphology. Images were taken from the retinal periphery at P8. (**H**) Quantification of microglia PUA at P5 and P8. (**I**) Light-sensitive photoreceptors: red/green and blue opsin identified rods (green) and rhodopsin identified cones (red), with nuclei in blue. (**J**) Relative quantification of rod and cone signals at P8. All representative images were taken at P8, except where indicated at P5. Scale bars: 100 μm in **A**; 50 μm in low-power images and 25 μm in high-power inset images in **C**; 25 μm in **E**; 50 μm in **G**; 25 μm in **I**. **B**, **D**, and **H** were analyzed by 1-way ANOVA with Tukey’s post hoc test; **F** and **J** were analyzed by unpaired 2-tailed Student’s *t* test. The field of view (area) for assessment of cell density was 6,250 μm^2^. Data represent mean ± SEM, with individual data points displayed. *N* ≥ 3 eyes from at least 3 mice for each group. *P < 0.05, **P < 0.01, ***P < 0.001, ****P < 0.0001.

In order to determine whether the severe defects in vascularization were associated with any damage to, or loss of, neuronal population of the retina, we visualized and quantified the neural retina in SMA mice. The neural retina is a deep, multilayered structure. Initial quantification of H&E-stained sections of whole retinas from SMA mice at P5 and P8 (Figure 4A) showed a significant reduction in retinal thickness in SMA retinas compared with controls at P5 (control, 162.8 ± 5.8 μm; SMA, 138.2 ± 1.3 μm; *P* < 0.01) and P8 (control, 132.8 ± 3.1 μm; SMA, 89.0 ± 3.0 μm; *P* < 0.001) (Figure 4B). Note that retinal thickness decreased in both control and SMA retinas between P5 and P8 (*P* < 0.001; Figure 4B). A further detailed immunohistochemical analysis of the different cells that make up the multilayered retina showed that numbers of retinal ganglion cells (RGCs) identified by BRN3a staining (Figure 4C) were similar at P5 in SMA and control littermate mice (control, 4.44 ± 0.4; SMA, 4.77 ± 0.3; *P* = NS) but significantly reduced in SMA at P8 (control, 3.7 ± 0.2; SMA, 1.9 ± 0.2; *P* < 0.001) (Figure 4D). There was no change in numbers of RGCs in control retinas between P5 and P8 (*P* = NS), while SMA retinas showed a significant loss of RGCs over the same period (*P* < 0.001) (Figure 4D). Next, focusing on P8, we stained amacrine and horizontal cells with PAX6 (Figure 4E), which revealed a significant reduction in SMA retinas compared with healthy littermate controls (control, 21.4 ± 0.9; SMA, 9.6 ± 1.1; *P* < 0.0001) (Figure 4F).

Microglia are implicated in both vessel and synaptic remodeling as well as in disease processes in the retina (39), and we had seen their apparent increase in SMA mouse retinas in our original observations (Figure 2A). We therefore quantified the microglia in whole-mount preparations, stained with GSL I/IB4 lectin and identified by their morphology (Figure 4G). Numbers of microglia were not changed in SMA retinas at P3 in comparison with control littermates (control, 13.0 ± 1.07; SMA, 19.8 ± 1.7; *P* = NS) but significantly increased at P5 (control, 10.9 ± 1.2; SMA, 19.7 ± 1.9; *P* < 0.05) (Figure 4H). By P8 microglia were almost absent in control retinas but were dense in SMA retinas (control, 2.0 ± 0.9; SMA, 32.8 ± 3.7; *P* < 0.001) (Figure 4H). Over this period, numbers of microglia were consistent between P3 and P5 in control retinas (*P* = NS) but decreased between P5 and P8 (*P* < 0.05). In SMA retinas, microglia were again stable between P3 and P5 (*P* = NS) but increased between P5 and P8 (*P* < 0.001) (Figure 4H). We also confirmed the identity of these cells using a second established marker, ionized calcium-binding adaptor molecule 1 (Iba1) (40). This showed the same pattern of increases in microglia in SMA at P8, with the appearance of large numbers of microglia in the deeper synaptic inner and outer nuclear layers in sections of the retina (Supplemental Figure 2). Conversely, in control retinas, microglia were noticeable by their absence in these deeper layers.

Finally, we examined the light-sensitive photoreceptors of the retina, where we identified rods by red/green and blue opsin staining and cones by rhodopsin staining at P8 (Figure 4I). Photoreceptors were depleted in SMA retinas compared with control littermates (control rods, 8.1% ± 0.6%; SMA, 0.9% ± 0.3%; *P* < 0.001; control cones, 23.4% ± 2.5%; SMA, 9.2% ± 3.1%; *P* < 0.01) (Figure 4J).

The loss of these retinal cells likely underlies the dramatic thinning of the retina that we initially observed in Figure 4A. These data show a close association between an early reduction in CNS vascularity and a concurrent or later loss of neurons. In turn, this is associated with a progressive increase in microglia.

### Endothelial injury markers are increased in peripheral blood from SMA patients.

To gather further insight into the microvascular system, we investigated the markers of endothelial injury in blood circulation in SMA patients. Vascular health is determined by a balance between endothelial injury and repair (41–43). In response to chronic vascular inflammation or trauma associated with endothelial injury, endothelial cells detach from vessel walls and enter the blood circulation (44–46). These circulating endothelial cells (CECs) allow vascular injury to be tracked in patients with vasculopathy (47–49).

**Figure 5:**
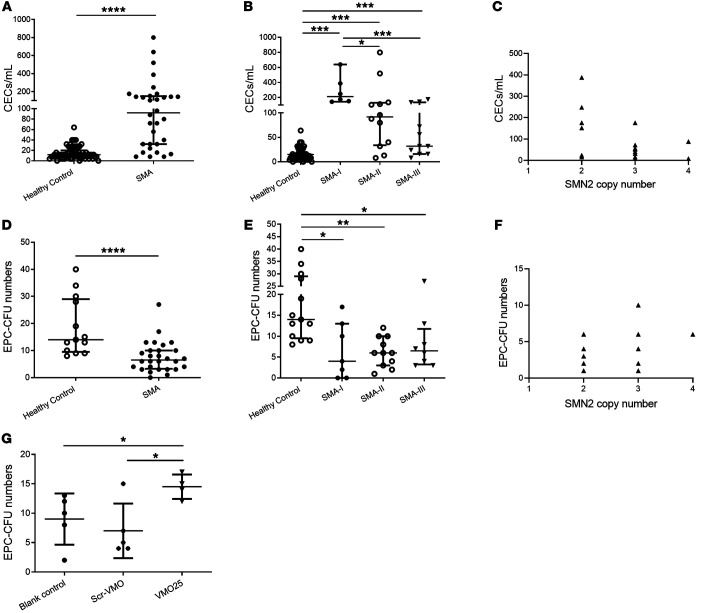
Increased vascular injury and decreased vascular repair revealed in peripheral blood from SMA patients. (**A**) Levels of CECs (number/mL) in peripheral blood from SMA patients (including type 1, 2, and 3, *n* = 32) and healthy controls (*n* = 17). (**B**) Comparison of CEC counts between healthy control (*n* = 17) and SMA type 1 (*n* = 6), type 2 (*n* = 12), and type 3 (*n* = 11). (**C**) Correlation between CEC counts and the copy number of *SMN2* gene in SMA patients (*r*^2^ = 0.2344, *P* < 0.05). (**D**) Comparison of EPC-CFU numbers in peripheral blood from SMA patients (including type 1, 2, and 3, *n* = 28) and healthy controls (*n* = 13). (**E**) Comparison of EPC-CFU numbers between healthy controls and SMA type 1 (*n* = 7), type 2 (*n* = 11), and type 3 (*n* = 8). (**F**) Correlation between numbers of EPC-CFUs and *SMN2* copy (*r*^2^ = 0.1696, *P* = 0.1435). (**G**) Comparison of numbers of EPC-CFUs from healthy controls (*n* = 13), SMA type 1 patients (SMA-I, *n* = 5), EPCs isolated from SMA type 1 and treated with scrambled Vivo-Morpholino (SMA-I+Scr-VMO, *n* = 5), and EPCs isolated from SMA type 1 and treated with therapeutic AON (SMA-I+VMO25, *n* = 5). **A** and **D** were analyzed by unpaired 2-tailed Student’s *t* test; **B**, **E**, and **G** were analyzed by 1-way ANOVA with Tukey’s post hoc test; **C** and **F** were analyzed by Spearman’s rank correlation coefficient for associations of CECs and EPCs with *SMN2* copy numbers. Data represent mean ± SEM. *P < 0.05, **P < 0.01, ***P < 0.001, ****P < 0.0001.

Therefore, to assess whether vascular damage was present in patients with SMA, the levels of CECs in peripheral blood were measured and compared with those in age-matched healthy controls. The CEC count in patients with SMA (*n* = 32) was higher, at 147/mL (8 to 800) compared with 15/mL (0 to 64) in healthy controls (*n* = 67, *P* < 0.0001) (Figure 5A). Significant differences in the CEC count were also detected in SMA patients with differing clinical severity when compared with healthy controls: type 1 SMA patients, 291/mL (144–640, *n* = 6, *P* < 0.0001); type 2 patients, 169/mL (8–800, *n* = 12, *P* < 0.0001); and type 3 patients, 64/mL (8–176, *n* = 11, *P* = 0.3111). Significant difference was also detected between SMA type 1 and type 3 patients (*P* < 0.0001; Figure 5B). Moreover, a significant negative correlation was found between the CEC count and *SMN2* copy number (*r*^2^ = 0.2344, *P* < 0.05) (Figure 5C).

These results provide evidence of an ongoing endothelial injury in SMA patients and suggest a close association between endothelial injury and disease severity and/or *SMN2* copy number. These findings also highlight the potential utility of CEC counts in peripheral blood as a novel cellular biomarker for SMA-associated vasculopathy.

### The potential of endothelial repair is decreased in SMA patients.

In the presence of vascular damage, concurrent recruitment of bone marrow–derived endothelial progenitor cells (EPCs) is an important mechanism for ongoing endothelial repair (43, 50). To assess this in SMA patients, we next carried out colony-forming unit (CFU) assays in angiogenic medium, a specific enumeration system for EPCs (51). We found a significant decrease in the number of CFUs from EPCs isolated from SMA patient blood samples (8 CFU/well, range 1–27, *n* = 28) compared with age-matched healthy control (19 CFU/well, range 8–40, *n* = 13, *P* = 0.0002) (Figure 5D). There was, however, no significant difference in the EPC-CFU count between different SMA subtypes: the reduction in EPC-CFUs was similar between SMA type 1 (6 CFU/well, range 0–17, *n* = 7), type 2 (6 CFU/well, range 1–12, *n* = 11), and type 3 patients (9 CFU/well, range 3–27, *n* = 8) (Figure 5E). Further, no correlation between the EPC-CFU count and *SMN2* copy numbers was detected (*r*^2^ = 0.1696, *P* = 0.1435) (Figure 5F).

This result indicates a decreased potential for endothelial repair in SMA patients. The lack of correlation between *SMN2* copy number and the number of EPC-CFUs indicates that a decreased potential for endothelial repair might be a general phenomenon in a range of SMA patients. Taken together, SMA patients show defective microvascular networks, increased vascular injury, and a reduced capacity for vascular repair, consistent with a generalized microvasculopathy.

### Antisense treatment ameliorates vascular repair defects in EPCs isolated from SMA patients.

As intrathecal delivery of the AON nusinersen is used for treatment in SMA, and we observed that systemic AON treatment was able to rescue retinal vascularity in SMA mice, we next set out to investigate whether AON treatment was able to rescue SMA-associated vasculopathy in vitro. We tested the effect of SMN-enhancing AON treatment on the defective EPC function in SMA patients. We treated patient-derived EPCs with a 25-mer AON (same sequence as used in mouse retina studies above) targeting the intronic splicing silencer N1 element (ISS-N1) in *SMN2* intron 7 (21). To avoid the potential confounding effect of transfection reagents, this AON was synthesized using Vivo-Morpholino chemistry (VMO25), as described previously (21). EPCs isolated from type 1 SMA patients (SMA-I, *n* = 5) were treated with VMO25 (SMA-I+VMO25) at 1 μM for 7 days in the EPC-CFU assay. VMO25 significantly increased the number of EPC-CFUs approximately 2-fold (15 CFU/well, range 12–17) in comparison with scrambled Vivo-Morpholino–treated (SMA-I+Scr-VMO; 7 CFU/well, range 4–15, *P* < 0.05) and untreated patients’ EPCs (9 CFU/well, range 2–13, *P* < 0.05) (Figure 5G). This suggests that to ameliorate systemic SMA vasculopathy, the systemic administration of AON is required.

### Endothelial cell–autonomous defects in angiogenesis drive the vascular phenotype.

**Figure 6:**
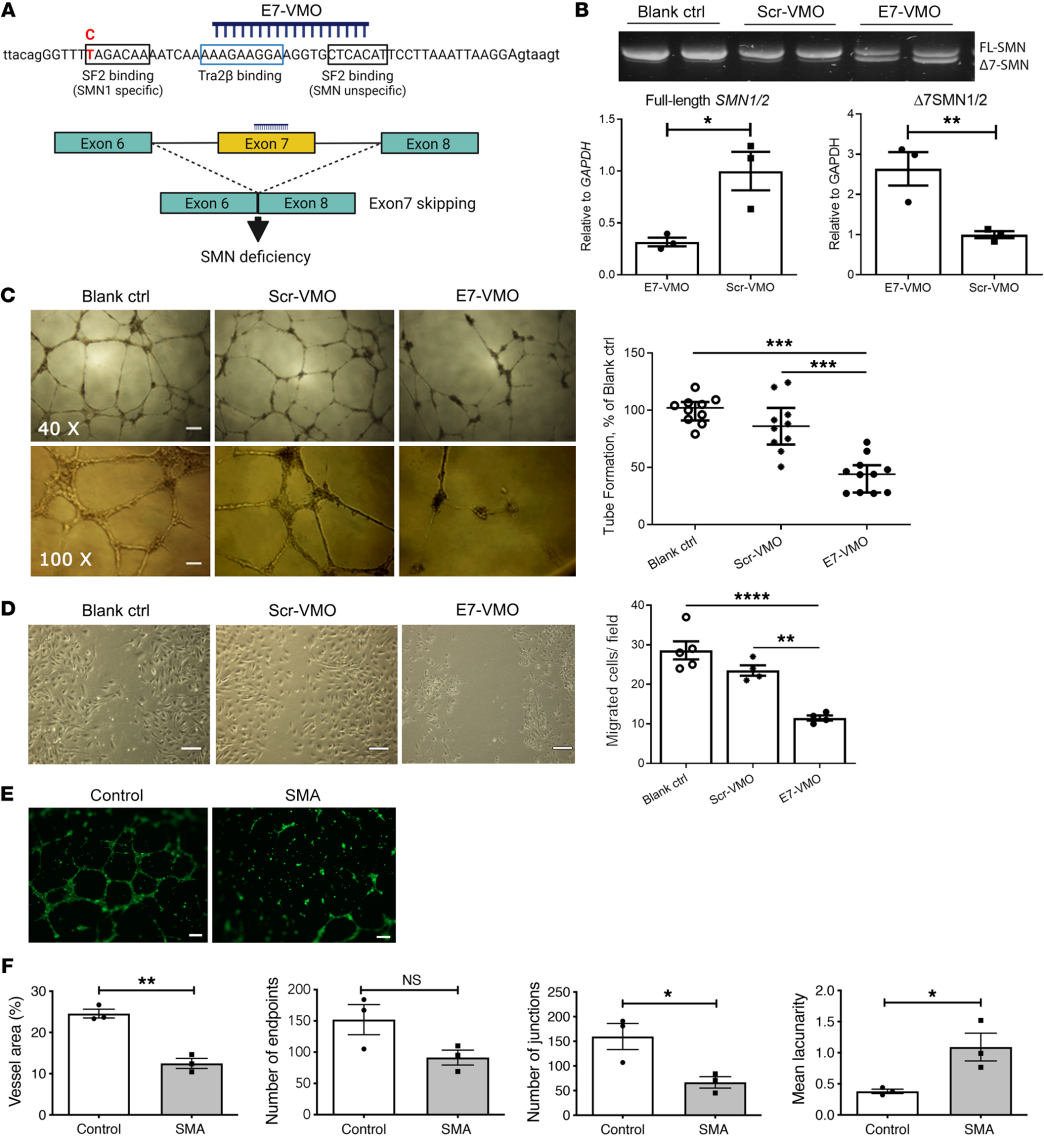
Defects in angiogenesis in cultured human endothelial cells with induced SMN deficiency. (**A**) AON was designed to target exon 7 in *SMN1* and *SMN2* genes to induce exon 7 skipping. (**B**) HUVECs were treated with exon 7–skipping Vivo-Morpholino (E7-VMO) or scrambled Vivo-Morpholino (Scr-VMO) and compared with untreated HUVECs (Blank ctrl). The *SMN1* and *SMN2* exon 7 skipped by AONs was measured by reverse transcriptase PCR and quantitative RT-PCR, respectively. Data were analyzed by unpaired 2-tailed Student’s *t* test. (**C**) Vascular tube formation in untreated HUVECs (blank control, *n* = 10), and HUVECs treated with Scr-VMO (*n* = 10) or E7-VMO (*n* = 11). Images were captured at an objective of ×40 and ×100, respectively. Tube formation was quantified as percentage of blank control. Scale bars: 100 μm in ×40 and 200 μm in ×100 images. Data were analyzed by 1-way ANOVA and Tukey’s post hoc test. (**D**) Endothelial cell migration in HUVECs of blank control, and cells treated with Scr-VMO and E7-VMO. HUVEC migration was quantified and analyzed by 1-way ANOVA and Tukey’s post hoc test. Scale bars: 200 μm. (**E**) Cultured endothelial networks from endothelial cells isolated from aortae harvested from SMA mice and healthy controls at P4–P6, visualized after calcein dye uptake. Scale bars: 200 μm. (**F**) Parameters on endothelial networks were analyzed by AngioTool. Data were analyzed by unpaired 2-tailed Student’s *t* test. Data represent mean ± SEM, with individual data points displayed. *P < 0.05, **P < 0.01, ***P < 0.001, ****P < 0.0001.

The findings detailed above all point to defects in angiogenesis, increased degeneration, and poor regeneration responses in the microvascular system of SMA patients. We therefore wanted to establish whether this represents a cell-autonomous endothelial phenotype, secondary to low levels of SMN protein. To understand the nature of the association between SMN and defective vascularity, we first performed a series of in vitro studies on angiogenesis in cultured human umbilical vein endothelial cells (HUVECs). As these are human cells carrying both *SMN1* and *SMN2* genes, we used an 18-mer exon 7–skipping AON to reduce SMN protein levels (Figure 6A) (52). This exon-skipping AON binding to both *SMN1* and *SMN2* pre-mRNA was synthesized using Vivo-Morpholino chemistry (E7-VMO), as above. After 48 hours of incubation, 1 μM E7-VMO induced approximately 70% exon skipping in *SMN1* and *SMN2* in HUVECs compared with scrambled control (Scr-VMO), as measured by quantitative reverse transcriptase PCR (Figure 6B; see complete unedited blots in the supplemental material).

We then used this E7-VMO to deplete SMN in HUVECs and investigate microvascular network formation and cell migration. HUVECs were initially incubated in Matrigel for 24 hours at 37°C, followed by treatment with E7-VMO or Scr-VMO at 1 μM for 48 hours. HUVECs treated with E7-VMO showed significantly (~50%) reduced HUVEC capillary network formation as compared with untreated (blank control) and Scr-VMO–treated HUVECs (Figure 6C).

The ability of endothelial cells to migrate, which is key to angiogenesis, was measured by the scratch migration assay (53). HUVEC migration was significantly reduced after E7-VMO treatment (11.5 ± 0.65, *n* = 4) compared with Scr-VMO control (23.5 ± 1.32, *n* = 4, *P* = 0.002) and blank control (28.6 ± 2.30, *n* = 5, *P* < 0.0001) (Figure 6D). These data suggest that a depletion of SMN protein in cultured endothelial cells results in defective tube formation and migration, both essential components of angiogenesis. As this effect is directly linked to SMN deficiency, we concluded that this represents a cell-autonomous defect.

We further confirmed the occurrence of the endothelial cell–autonomous defects in cells derived from SMA mice. Endothelial cells were isolated from the aorta of SMA mice and control littermates at P4–P6 and placed into culture. Cells were grown in Matrigel for 16 hours to test their ability to form tubes. SMA endothelial cells showed significantly reduced tube formation (Figure 6E). The percentage of the total area covered by vessels was reduced by approximately 50% (control, 24.57 ± 1.06; SMA, 12.49 ± 1.22; *P* < 0.01); there was less branching, as the number of junctions was reduced by approximately 60% (control, 159.7 ± 26.49; SMA, 66.7 ± 11.4; *P* < 0.05), while lacunarity (the mesh size of the spaces in the vessel network) was approximately 3 times greater in control (1.09 ± 0.22) compared with SMA (0.38 ± 0.03; *P* < 0.05) cultures. Although not to a statistically significant degree, SMA cultures also tended to have fewer endpoints (tubes terminating in a growing tip) (control, 152 ± 24.01; SMA, 91.3 ± 11.98; *P* = 0.08) (Figure 6F). Taken together, these data confirm an endothelial cell–autonomous defect in response to reduced SMN protein levels resulting in reduced angiogenesis.

## Discussion

In this study, we have identified and characterized microvascular defects in SMA, investigated by imaging, histological, molecular, and cellular studies in SMA patients, transgenic SMA mice, and cellular models with SMN deficiency. We reveal a widespread microvascular pathology that is amenable to systemically delivered SMN-restoring therapy, and describe gross and cellular biomarkers of vascular pathology.

The eye and neural retina are a window on the brain, and an area of growing interest in neurodegenerative diseases (29, 54, 55), including motor neuron disease (56). However, retinal microvasculature has not previously been examined in SMA, even though there are changes in CNS neuronal and vascular parameters in SMA (22, 28). Here, by analyzing ultra-widefield ophthalmoscopy images, we were able to detect a retinal vascular phenotype in children with SMA. Although the long-term biological implications of this finding in children are unknown, a lower FD in the retina implicates subclinical microvascular changes in the systemic vasculature (57, 31). While FD is a promising measure for capturing the state of the vascular geometry, there are limitations to its computation using the method of automatic vessel detection or segmentation. We therefore applied 2 vessel segmentation methods to evaluate the stability of observations of statistically significant changes in FD. The key difference between the 2 techniques is the amount of vasculature that is detected. The Pellegrini technique (previous version of VAMPIRE-UWF) performs well in segmenting the larger and more prominent vessels, whereas the IterNet technique (current version of VAMPIRE-UWF) segments both the prominent and the smaller vessels. Therefore, the IterNet technique includes more of the vasculature in FD computation than the Pellegrini technique. We believe the influence of including a more complete detection of the vasculature in FD computation is the contributing factor to a significant difference in the posterior ROI that was not observed with the Pellegrini technique, as this region contains numerous small vessels. In addition, recent work reported a non-uniform FD (obtained from Pellegrini segmentation) in the 4 retinal quadrants and a decrease in FD with increasing distance from the fovea in ultra-widefield retinal images from healthy people (58); therefore, a difference between ROIs is not unexpected. However, it is not clear how this may relate to specific clinical vulnerability of different retinal regions that may have different metabolic demand for perfusion.

In SMA mice, widespread tissue hypoxia and multiorgan cellular hypoxic response have been previously demonstrated in both CNS and periphery (28). This was accompanied by increased glucose uptake in many affected organs, including spinal cord and eyes, as part of the hypoxia response (28). Hypoxia response is usually started by hypoxia-inducible factor 1 (HIF-1) and HIF-2 (59). However, no upregulation of HIF-1α or HIF-2 protein was detected in either of the hypoxic organs in SMA mice at the time point of the analysis (Supplemental Figure 3), suggesting that tissue hypoxia is a rather complex dynamic cellular process under SMN deficiency. We have nevertheless shown that the widespread tissue hypoxia is associated with increased neuronal vulnerability to hypoxia in SMA mice (28). It is hypothesized that a disrupted neurovascular unit, consisting of local neurons, astrocytes, and vascular endothelial cells, could exist in SMA (60). Indeed, the disruption of neuron-astrocyte-endothelial communication has recently been reported in both Alzheimer’s disease (31, 61) and ALS (62). Early capillary regression results in insufficient local capillary blood flow and hypoxia (22), and we suggest that the reduced vascular tissue perfusion might in turn accelerate neuronal loss, leading to a vicious cycle involved in SMA disease progression. Further, defects in astrocyte-neuron communication in SMA (63) may also affect astrocyte–retinal ganglion cell interactions in the retina. Finally, microgliosis was initially reported in SMA mouse ventral horn (64), the effect of SMN depletion in microglia has been highlighted (65), and microglia are identified by GSL I/IB4 stain following ischemia (66). These observations suggest an intrinsic and an extrinsic origin for microgliosis in SMA, which is particularly associated with synapse loss. This fits with our observation of an increase in microglia observed in the deeper layers of the retina, where neurons are lost. These observations suggest that the microgliosis seen in SMA mouse retinas may be indicative of a more broadly relevant phenotype in SMA.

Although we could not perform retinal imaging in type 1 SMA infants because of the positioning compliance needed, we anticipate that the retinal vasculature abnormalities are likely to be present also in this severe form of SMA. The alterations in retinal vasculature in the children with milder SMA types 2 and 3 are in keeping with the nature of the reported anomalies in retinal vasculature in SMA mouse models, though mice develop their vasculature postnatally, in contrast to human infants, who have retinal vasculature reaching the retinal periphery by term (67).

The identification of the retinal vascular phenotype is timely as detailed ocular surveillance has recently been put in place for SMA patients receiving risdiplam, due to the observation of retinal toxicity in preclinical toxicology studies (68). Reassuringly, neither the photoreceptor degeneration nor microcystoid macular degeneration previously detected in treated monkeys were seen in risdiplam-treated patients (69). The study did include fundus photography, but the vascularity of the retina was not analyzed. The less complex and less dense vasculature we quantified in SMA patients may not be obvious by direct inspection of an individual fundus image without FD analysis. Further analysis of the vasculature of these retinal images may provide more valuable information in this patient group. Future work to assess the extent of the microvascular structure defect in other anatomical localization — for example, the nailbed capillaries, which can be assessed noninvasively using a capillaroscopy system (70, 71) — would also be of interest.

Our studies demonstrate that there is an imbalance in endothelial injury and repair in SMA, as indicated by increased numbers of CECs and decreased numbers of EPCs in SMA patient blood (Figure 5). CECs have been used as a marker to track endothelial injury in a wide range of acquired vascular disorders (46, 72), but never in SMA. We demonstrate that in SMA patients there was a correlation between the *SMN2* copy number and the increased CEC number, indicating the potential of CEC concentration in blood as a cellular biomarker of endothelial injury in SMA (Figure 5). Bone marrow–derived EPCs play an important role in endothelial maintenance and vascular healing (50), and act as a cellular biomarker of endothelial repair in various vascular diseases (43, 51, 73). The decreased EPC-CFU numbers in SMA patients (Figure 5, D and E) and the sensitive response of cultured EPCs isolated from SMA type 1 patients to AON treatment in vitro (Figure 5G) further support EPCs as a potential cellular marker indicative of endothelial repair in SMA.

Finally, we revealed defective angiogenesis in cultured SMN-depleted endothelial cells, indicative of a cell-autonomous, SMN-dependent pathology. While cell-autonomous defects secondary to SMN deficiency have been reported in key components of the motor system, including motor neurons (74), skeletal muscle satellite cells (75), and Schwann cells (76), there are no data on the vascular system. We demonstrate that cultured human endothelial cells with induced SMN deficiency display defects in vascular tube formation and endothelial cell migration (Figure 6, A–D), but not endothelial cell apoptosis (Supplemental Figure 4). The cell-autonomous defect was further confirmed in endothelial cells isolated from the aorta of SMA mice, which also showed reduced vascular tube formation (Figure 6, E and F), a surrogate for angiogenesis. These data confirm the angiogenic phenotype described in the mouse retinal data, where the amount of outgrowth and number of growing tips of the vascular plexus were both depleted (Figure 2). In addition, the absence of vessel loss, failure of vessels to mature, and disorganized vessel growth all point toward a primary failure in the ability of endothelial cells to grow and develop.

Our study indicates that microvasculopathy is a widespread phenomenon in patients and mice affected by SMA. This microvasculopathy is driven by an endothelial cell–autonomous defect in angiogenesis. This likely accelerates disease progression by further compromising organs that are already affected by SMN deficiency. Our findings emphasize the importance of therapeutic intervention to address the peripheral manifestation of SMA in addition to the CNS. While some therapies target both the periphery and CNS (10, 68), their effect on the vascular phenotype is still unknown. Onasemnogene abeparvovec has shown efficacy in restoring motor function and survival in type 1 SMA patients, especially when administered close to disease onset (10). However, its limitation in rescuing the vascular-related clinical features (digit necrosis and diffuse macular rash) was highlighted in a recent report of a child with SMA type 0 who was treated with both nusinersen and onasemnogene abeparvovec. These peripheral manifestations remained unchanged, despite the modest motor improvement (77). While AAV9 serotype is efficient in crossing the blood-brain barrier, it induces minimal transduction of endothelial cells, while maintaining the capacity to transduce neurons after the endothelial transcytosis (78). Relevantly, thrombotic microangiopathy (TMA), characterized by arteriole and capillary endothelial pathology and microvascular thrombosis, is a severe adverse effect recently reported in several SMA infants treated with onasemnogene abeparvovec (79). While the exact mechanism of this adverse reaction is still unknown, suggested etiologies include direct AAV toxic effect (80) or immune-mediated reactions to AAV vector (81). Our finding provides a further explanation and indicates that the underlying endothelial dysfunction in SMA may predispose some patients to TMA following systemic AAV gene therapy. To reduce the incidence of TMA, it would be important to evaluate the baseline vascular status of patients before commencing AAV-mediated treatment, as this might help to identify potentially susceptible individuals.

Taken together, our data identify microvasculopathy as a fundamental feature of SMA, which is driven by reversible autonomous endothelial cell defect. Future studies on endothelial cell–specific SMN restoration in SMA mice will be needed to better understand the role of endothelial cells in disease pathogenesis and progression, and to what extent that microvasculopathy may contribute to the multiorgan involvement in SMA. In light of all these findings, our study suggests that therapeutic strategies for SMA should also include the correction of the SMN deficiency in the periphery, including the vascular system.

## Methods

### Patients and controls.

All studies were performed in children with SMA with different levels of clinical severity attending the Great Ormond Street Hospital NHS Foundation Trust, London, during October 2015 and February 2018. Parental consent was obtained for all children involved in the study, which was approved by the national ethics committees (see below). Inclusion criteria for children with SMA were as follows: age less than 18 years, and a diagnosis of SMA confirmed by genetic diagnosis indicating a genomic deletion in the *SMN1* gene. Control samples were obtained from healthy sex- and age-matched children.

### SMA mice and procedures.

SMA transgenic mice, FVB.Cg-Tg(*SMN2*)_2_Hung *Smn1*^tm1Hung^/J, also called the Taiwanese model (35), were initially purchased from The Jackson Laboratory (TJL005058). Mice were bred and experimental procedures were carried out in the Biological Service Unit, University College London, in accordance with the Animals (Scientific Procedures) Act 1986.

Newborn SMA mice were subcutaneously injected with a single dose of PMO25 at 40 μg/g. Untreated SMA control mice were injected with a similar volume of saline as previously described (21).

### Antisense oligonucleotides.

The therapeutic AON PMO25 and the *SMN1/2* exon 7–skipping Vivo-Morpholino (E7-VMO) were purchased from Gene Tools. The antisense sequences are listed in Supplemental Table 1.

### Human retina imaging and data analysis.

An ultra-widefield (UWF) scanning laser ophthalmoscope (OPTOS California, version 2.4.6.1031, Optos plc.) was used to take non-dilated fundus images of the retinas of 11 type 2 (*n* = 6) and type 3 (*n* = 5) SMA patients, median age 11 years (range 6–16 years), and from 23 healthy controls, median age 9 years (range 3.5–17 years). A plot of the ages is presented in Supplemental Figure 5. These images were centered on the fovea with on-axis symmetry of 15 μm (TIFF format; 4,000 × 4,000 pixels). Measurements of the vasculature were obtained using specially designed software for analyzing UWF images (Vasculature Assessment and Measurement Platform for Images of the Retina [VAMPIRE], version VAMPIRE-UWF, Universities of Edinburgh and Dundee, United Kingdom [UK]).

Fractal dimension (FD) is a unitless measure between 1 and 2. It describes how a repeating pattern (such as the retinal vasculature) fills the space in which it is contained. FD is influenced by (a) the space or region of interest (ROI) in which it is measured (e.g., FD of a branching pattern may decrease if the space in which it is contained increases, i.e., it fills the larger space less); (b) accuracy of the vessel segmentation; and (c) image quality [which directly affects (b)]. We therefore standardized the ROI (provided by the VAMPIRE-UWF software) so that FD was computed for the same area within each image (denoted “standardized ROI”) (Figure 1). We only included images clear of eyelashes and eyelids, which can interfere with analysis, resulting in 21 images from SMA patient eyes and 46 control eyes. Next, the retinal vessels were automatically segmented from the background using 2 different methods (33, 34). The Pellegrini et al. 2014 method segments prominent vessels, and the IterNet 2020 method segments prominent and smaller branching vessels. The segmented images were then skeletonized (i.e., the center line of the vessel network was represented as 1-pixel-wide curved lines). The difference in vessel skeletons from the 2 different segmentation methods can be seen in Figure 1. The skeletons were inspected and any minor erroneous segmentations corrected manually. The standardized ROI (VAMPIRE-UWF) had an area of 319 mm^2^ and included the posterior pole, midperiphery, and a small portion of the far periphery. The standardized ROI was subdivided into a posterior ROI (an annulus centered on the optic disc that extends 3 optic disc diameters away from the optic disc boundary, also known as zone C) (82) and a midperiphery ROI (area between standardized and posterior ROI) to investigate regional changes in FD. FD was computed from the 3 regions using methods reported previously (83).

### Mouse retina dissection and immunohistochemical staining.

See Supplemental Methods.

### Quantification of mouse retinal vascularity.

AngioTool software was used for quantification of the retinal vasculature (https://ccrod.cancer.gov/confluence/display/ROB2/Home). For vessel outgrowth, a single figure was obtained for each retina. For endpoint number and lacunarity, a systematic and random method was used to capture ROIs for assessment, and thus each data point represents a single field.

### Quantification of mouse retinal thickness and retinal cells.

Stained slides of mouse retinas were imaged using a standard upright Nikon Eclipse E400 microscope. Images were captured using a QICAM Fast 1394 camera and Volocity imaging software (PerkinElmer). Retinal thickness was measured directly from calibrated images. Retinal cell density was calculated using a systematic, random methodology, based on a method reported previously (84). Additional details are presented in Supplemental Methods.

### Immunomagnetic bead extraction of circulating endothelial cells from peripheral blood.

Circulating endothelial cells (CECs) were extracted from whole blood by CD146-coated immunomagnetic beads (5050-P, BioCytex) using an international consensus protocol (45). The extracted CECs were counted using a Nageotte chamber under a fluorescence microscope and were defined as *Ulex europaeus* lectin (L9006, Sigma-Aldrich) bright cells that were greater than 10 μm in size, with 5 or more magnetic beads attached.

### Endothelial progenitor cell colony-forming units.

Peripheral blood mononuclear cells (PBMCs) were isolated by density centrifugation (Lymphoprep, Axis Shield). After purification with 3 washing steps, 2 × 10^6^ PBMCs were plated on fibronectin-coated 24-well plates. Cells were cultured and maintained in endothelial growth medium (EGM-2) supplemented with growth factors according to the manufacturer’s recommendations (PromoCell, Heidelberg, Germany), plus 20% FCS and 40 ng/mL of vascular endothelial growth factor (VEGF). After 4 days of culture, nonadherent cells were removed by washing with PBS.

To study the effects of AONs, 1 μM VMO25 or Scr-VMO was added to the medium at day 4. Culture medium was changed to maintain the cells in culture until day 7. The numbers of endothelial progenitor cell colony-forming units (EPC-CFUs), characterized by a cluster of cells surrounded by elongated spindle-shaped cells, were counted manually in a minimum of 2 wells in 24-well plates by 2 independent observers who were unaware of the experiment design. Results were presented as average number of EPC-CFUs per well.

### HUVEC cultures and induced SMN1 and SMN2 exon 7 skipping by Vivo-Morpholino.

See Supplemental Methods.

### HUVEC tube formation and cell migration assay.

See Supplemental Methods.

### SMA mouse endothelial cell cultures and tube formation assay.

See Supplemental Methods.

### PCR, real-time PCR, and Western blotting.

See Supplemental Methods.

### Statistics.

For data collected from SMA patients and controls, numeric results were summarized as median and range. The D’Agostino and Pearson omnibus normality test was used to examine overall differences in experimental laboratory markers between the study groups, followed by the 2-tailed Mann-Whitney *U* test. Associations of CECs and EPCs with *SMN2* copy numbers were assessed using Spearman’s rank correlation coefficient.

Statistical analysis of human retinal parameters was conducted using generalized estimation equations (GEEs) (R version 3.6.0, geepack, ref. 85; and ref. 86) that account for the correlation between both eyes of an individual. As GEEs are sensitive to outliers, extreme values were imputed to the mean of the group (87).

All in vitro experiments were performed in triplicate unless otherwise stated, and values are presented as mean ± SEM unless otherwise specified. Statistical differences between 2 groups for in vitro experiments and in vivo studies in mice were determined by unpaired 2-tailed Student’s *t* test; statistical analysis in more than 2 groups was performed by 1-way ANOVA followed by post hoc Tukey’s test. A *P* value less than 0.05 was considered significant. All analysis was performed using GraphPad Prism software.

### Study approval.

The study was approved by the national ethics committees, including the West London & GTAC Research Ethics Committee (REC reference 06/Q0406/33), National Research Ethics Service (NRES) Committee London–Camberwell St Giles (REC reference 13/LO/1894), and NRES Committee London–Bromley (REC reference 13/LO/1748). Blood samples were supplied by the Medical Research Council (MRC) Centre for Neuromuscular Diseases Biobank London (http://www.cnmd.ac.uk). All parents provided written, fully informed consent and affected children their assent if older than 5 years, prior to inclusion in the study. All participants were anonymous in this study. Experiments on animals were performed under Home Office project licenses PP2611161 and P92BB9F93. All treatment procedures conducted in mice were approved by UK Home Office before being carried out in the Biological Services Unit, University College London Great Ormond Street Institute of Child Health, in accordance with the Animals (Scientific Procedures) Act 1986.

## Author contributions

HZ, YH, PB, THG, DAT, SHP, and FM conceived and designed the research studies. HZ, YH, AT, DAT, EP, TM, EHG, M Sanson, FC, GH, HKS, TN, JM, QZ, and AH conducted the experiments and analyzed the data. M Scoto and GB provided clinical samples and data. HZ, SHP, THG, KH, PB, DAT, and FM wrote the manuscript. HZ, YH, M Scoto, AT, and EP agreed to share first authorship due to their important contributions and heavy involvement in conducting the experiments, analyzing the data, and presenting the results. The order of these authors was decided following transparent discussion in which these parameters were taken into account: involvement in the study since its inception, including study design; the contribution on multiple fields relevant for authorship; and the intensity of the work performed for the manuscript.

## Supplementary Material

Supplemental data

## Figures and Tables

**Table 1:**
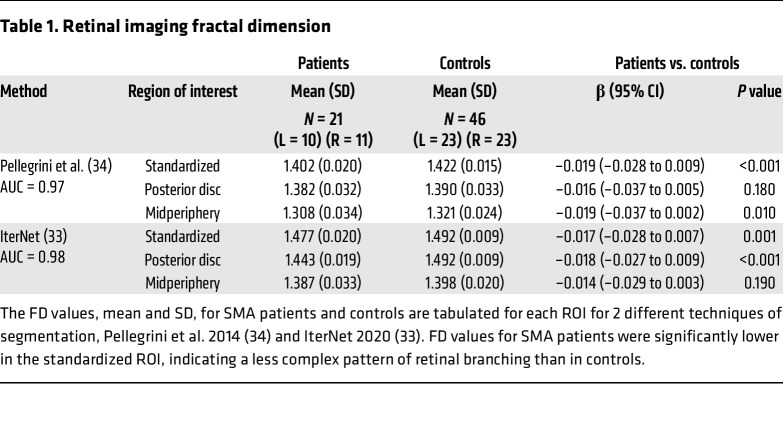
Retinal imaging fractal dimension

## Version 1 09/13/2022

In-Press Preview

## Version 2 11/01/2022

Electronic publication
