# Supplementary material for: Microvasculopathy in spinal muscular atrophy is driven by a reversible autonomous endothelial cell defect
Source: J Clin Invest. 2022 Nov 1;132(21):e153430. doi: 10.1172/JCI153430 (PMC9621128; doi:10.1172/JCI153430)
Supplement: Supplemental data [file jci-132-153430-s062.pdf]

Supplemental Data

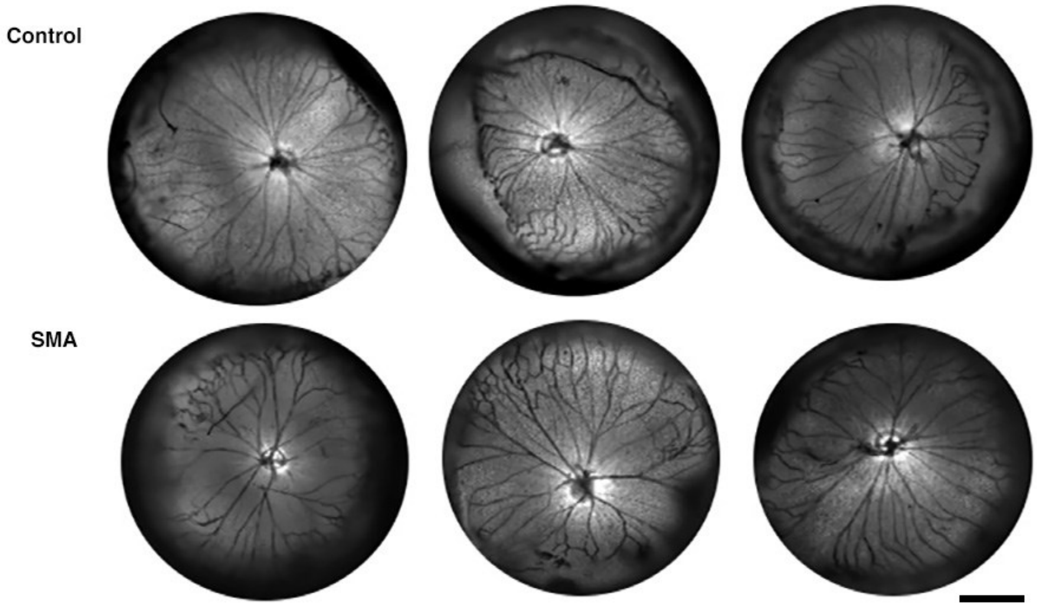

Supplemental Figure 1 The pre-existing hyoid vasculature appears normal in SMA compared to control mice

Images at multiple focal plains were captured form dissected but intact retinas enucleated from P5 Taiwanese and control mice. After digital combination of image stacks to visualise the entire hyaloid vasculature, projected images of 3 sets of representative image pairs indicated no observable differences in the form or extent of the hyaloid. Scale bar = 500µm.

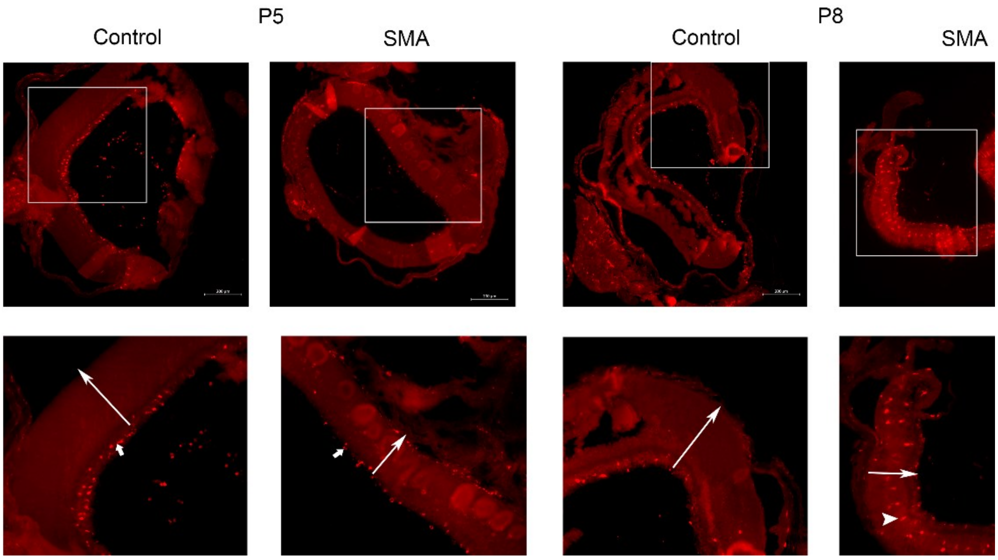

Supplemental Figure 2 Microglia in mouse retina are also identified by iba1 staining

Sections of SMA mouse retinas were stained with an antibody recognising iba1, a widely accepted marker for microglia. Appearances were similar to those obtained with GSL I/ IB4 stain. At P5, control and SMA retinas both showed small numbers of microglia located on the inner surface of the retina (arrows). At P8 control retinas showed a very similar appearance, with microglia located again on the inner retinal surface. However in SMA retinas at P8 the appearance was quite different with many more microglia located on the inner retinal surface, and also many located in the deeper (INL and ONL) layers of the retina. Long arrows indicate inner to outer layers of the retina, for clarity in high power images (lower lane, from the inset boxes in the upper lane). Scale bar = 200µm in low power and 100µm in high power images.

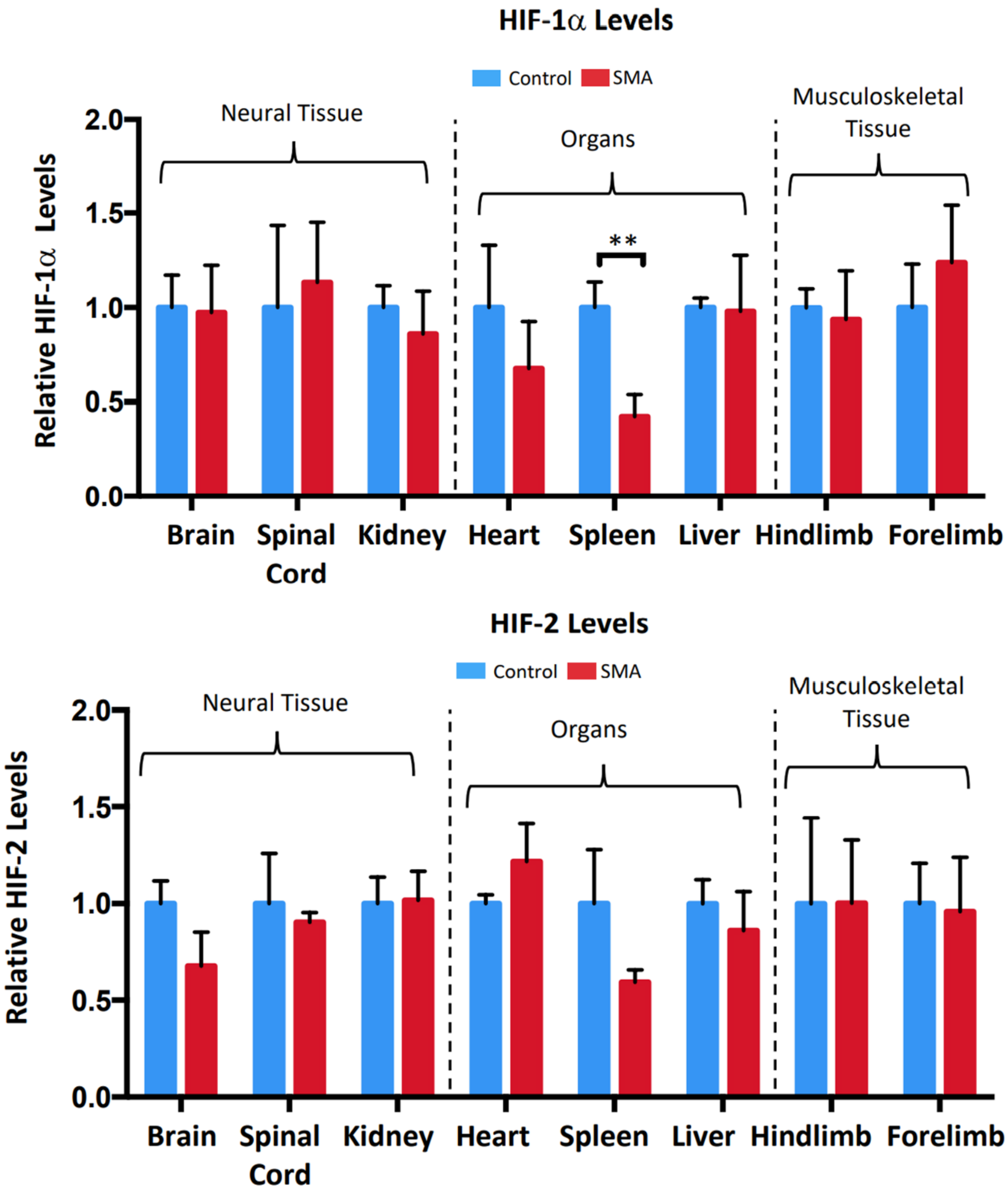

Supplemental Figure 3 HIF-1α and 2 levels are not upregulated in hypoxic SMA mice tissue

HIF-1α and HIF-2 levels were tested in brain, spinal cord, kidney, heart, spleen, liver, hindlimb and forelimbs, all tissues that had been shown to be hypoxic in SMA mice at D5. There was no significant difference between controls and SMA samples, except for spleen on HIF-1 α, where it was significantly lower in SMA mice compared to healthy littermate controls (**P<0.01, n=3/group).

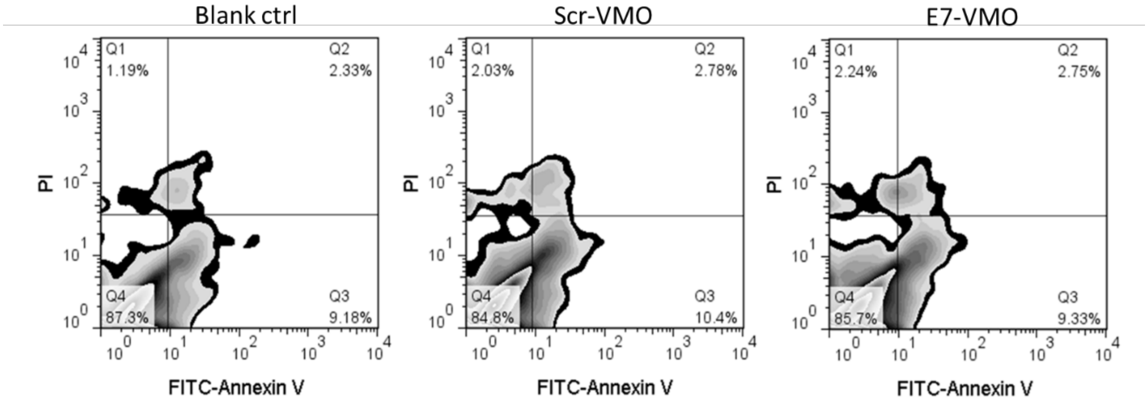

Supplemental Figure 4 Apoptosis assay of HUVECs.

Flow cytometry assay of FITC-Annexin V was performed in HUVECs treated with Scr-VMO, E7-VMO or blank control. The amount of apoptosis was determined as the percentage of Annexin V-positive cells/PI-negative cells (the upper right quadrant).

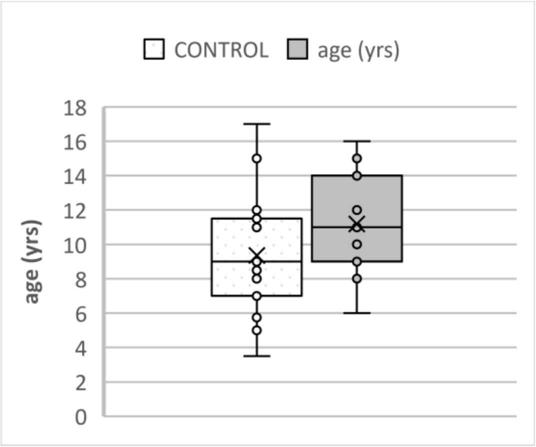

Supplemental Figure 5 Ages of SMA patients and healthy controls examined in retina imaging study.

Retinal images were captured in 11 SMA patients, median age 11 yrs (range 6-16yrs), and from 23 healthy controls, median age 9yrs (range 3.5-17yrs).

Supplemental Table 1 Sequences of the antisense oligonucleotides used in this study

Antisense oligonucleotide	Sequence	Chemistry
PMO25	5’-GTA AGA TTC ACT TTC ATA ATG CTG G-3’	Phosphorodiamidate morpholino
E7-VMO	5’- GTG AGC ACC TTC CTT CTT-3’	Vivo morpholino
Scr-VMO	5’- CTC ACG AGG TTC CTT GTT-3’	Vivo morpholino

Supplemental Table 2 Primary antibodies used in the Immunohistochemical staining of retinal vasculature in SMA mice

Antibody	Cat. No.	Dilution	Target Cell
GSL-Rhodamine	RL-1102 (Vector Labs)	1:100	Endothelial cells microglia
GSL-FITC	FL-1101 (Vector Labs)	1:100	Endothelial cells microglia
α-Smooth muscle actin (α -SMA)	Ab5694 (Abcam)	1:200	Smooth muscle/blood vessels
Collagen IV	Ab6586 (Abcam)	1:200	Basil lamina
S100	Ab868 (Abcam)	1:200	Astrocytes
PAX6	DSHB Pax6 (DHSB)	1:400	Horizontal and amacrine cells
Brn3a	MAB1585 (Millipore)	1:40	Retinal ganglion cells
Rhodopsin	O4886 (Sigma)	1:40	Rod photoreceptors
Red/Green Opsin	AB5405 (Millipore)	1:200	Cone photoreceptors
Blue Opsin	Sc-14363 (Santa Cruz)	1:200	Cone photoreceptors

Supplemental Table 3 **Sequences of primers used in this study**

Primer	Sequence (5’ to 3’)	Size
hSMN1/2	F: GTAAGATTCACTTTCATAATGCTGG<br>R: CTACAACACCCTTCTCACAG	505 bp (full-length)<br>451 bp (Δ7)
hFL-SMN1/2	F: ATACTGGCTATTATATGGGTTTT<br>R: TCCAGATCTGTCTGATCGTTTC	133 bp
hΔ7-SMN1/2	F: TGGACCACCAATAATTCCCC<br>R: ATGCCAGCATTTCCATATAATAGCC	125 bp
hGAPDH	F: TTGAGGTCAATGAAGGGGTC<br>R: GAAGGTGAAGGTCGGAGTCA	117 bp

Supplemental Methods

Mouse retinas dissection, fixation and cryo-sectioning

Retinas were dissected out of mouse enucleated eyes by piercing the edge of the lens with a syringe needle and cut carefully around the edge of the lens and remove it. The humour was removed using forceps, along with any visible hyaloid vasculature. The fibrous retinal pigment epithelium was gently cut away, exposing the inner retinal layer in which four or five radial incisions were made to achieve a ‘flower-shaped’ retina that could be whole-mounted and stained. Finally, retinas were fixed by slowly applying drops of -20ºC methanol over the tissue until it turned white, then the tissue was flooded with more methanol and left on ice for 20 minutes. Retinas were washed in 0.1M phosphate buffered saline (PBS) solution prior to staining.

For immunofluorescence staining, pre-fixed tissue was suspended in 30% sucrose solution in 0.1M PBS, embedded in OCT at -35˚C using a dry ice-isopentane slurry, sectioned at 8μm on a Leica cryostat and stored at -20˚C before staining. For H&E staining, pre-fixed tissue was embedded in paraffin wax and sectioned on a Leica microtome at a thickness of 8μm and mounted onto slides.

Immunohistochemical staining of mouse retinas

For wholemount retina staining, mouse retinas were permeabilized overnight at 4˚C in a blocking solution of 0.1M PBS containing 1% BSA and 0.5% Triton-X 100. The following day retinas were rinsed in PBS and washed twice in PBlec (PBS with 0.1mM $CaCl_2$, 0.1mM $MgCl_2$ and 0.1mM $mNCl_2$). Retinas were then incubated in a selection of antibodies, detailed below.

For retina section staining, slides were incubated in a blocking solution at room temperature for 1 hour. Primary antibodies (below) were added onto slides and incubated overnight at

4˚C. Slides were then washed in PBS and incubated in secondary antibodies for 2 hours at 4˚C. Slides were washed in PBS and mounted in MOWIOL (Sigma) mounting medium for imaging.

The primary antibodies were: Rhodamine (RL-1102) or Fluorescein (RL-1101) -conjugated GSLI (Vector Labs, 1:100), Alpha smooth muscle actin (Ab5694, Abcam, 1:200), Collagen IV (Ab6586, Abcam, 1:200), PAX6 (DSHB Pax6, Developmental Studies Hybridoma Bank, 1:400), Brn3a (MAB1585, Millipore, 1:40), Rhodopsin (O4886, Sigma, 1:40), Red/ Green Opsin (AB5405, Sigma, 1:40), Blue Opsin (sc-14363, Santa Cruz, 1:200). DAPI (Sigma) was used as a counterstain in sections to show nuclei. Secondary antibodies were either Donkey anti-goat Cy3 (AB6949, Abcam, 1:250) or Goat anti-rabbit Alexa Fluor 568 (AB175471, Abcam, 1:250). A summary of the antibodies was listed in Supplemental Table 2.

Quantification of mouse retinal thickness and retinal cells

Retinas were embedded, sectioned, and placed onto slides without specific alignment. A grid overlay was placed over these randomly oriented sections, and from the top left every 3rd grid square was counted (whole nucleus within or touching top and right-hand edges counted, but not counted if touching bottom or left-hand edges of sampling box). Data is expressed as cells PerUnit Area (PUA), where each grid square had an area of 6250μm^2.

HUVEC cultures and induced SMN1 and SMN2 Exon 7-skipping by vivo-morpholino

HUVECs (C-12200, PromoCell) were cultured in EGM-2 medium supplemented with 2 % FCS, hydrocortisone, fibroblast growth factor (FGF-2), VEGF, R3-insulin-like growth factor-1, epidermal growth factor, heparin, ascorbic acid, gentamycin and amphotericin B, as supplied by the manufacturer (C-22010, PromoCell) at 37 °C in 5 % CO2 in a humidified incubator. HUVECs at 80 % confluency (passages 2–5) were used for experiments.

Prior to AON treatment, HUVECs were trypsinized and plated in 6-well plates at the density of 2×10^5 cells/well. The vivo-morpholino to induce *SMN1* and *SMN2* exon 7 skipping (E7-VMO, Gene Tools) and scrambled vivo-morpholino (Scr-VMO) were then added into each well at 1 μM and incubated for 48 hours for further experiments.

HUVEC tube formation in matrigel assays

HUVECs were treated with either E7-VMO or Scr-VMO at 1 μM or blank control without VMO for 48 hours. Growth factor reduced Matrigel Matrix (Becton Dickinson Labware, Oxford, UK) was thawed and placed in 96-well plates at 37 °C for 30 min to solidify. Treated HUVECs were then seeded on matrigel-coated 6-well plates at a density of 5×10^3 cells/well in the endothelial cell growth medium and incubated in 5% CO_2 at 37°C for 18-22 hours. Examined the cells using a phase-contrast microscope and took 2-3 images from each well. The number of branches, a measure of the capability to form tubes and a reflection of the angiogenic ability, was counted in each well.

Endothelial cell migration - scratch assay

The migratory potential of HUVECs was assessed with a wound healing scratch assay. HUVECs were cultured until confluency in 6-well culture plates as described above. A scratch was made in the confluent monolayer with a P200 pipette tip. Plates were washed twice with PBS to remove any detached cells. HUVECs were then cultured in endothelial cells growth medium with or without AONs for a further 24 h. After the incubation, cells were washed twice with PBS gently to remove any detached or dead cells. The scratched area was photographed at t=0, and t=24 hours with a phase contrast microscope. The number of migrated cells that crossed into the scratched area were counted as a measure of the capability of endothelial cells migration.

SMA mouse endothelial cell cultures

Aortas were collected and cut longitudinally. The aorta pieces were placed on the collagen gel surface with the inner face down. Endothelial medium (211-500, Sigma-Aldrich) was added after 24h in the incubator. After 5-7 days when confluence was reached, aorta pieces were removed, collagen dissolved with 0.3% collagenase H and new medium added. The endothelial cells which had migrated into the collagen were maintained for culture.

SMA mouse endothelial cell tube formation assay

Mouse endothelial cells generated as above, were seeded into matrigel at 25,000 cells/well concentration at passage 2 and imaged after 16h. At this time point cells have begun to form into tubes, which were stained with 2µM calcein (ab141420, Abcam) for 30 minutes at 37ºC before imaging. Mean lacunarity (mesh size), total number of junctions (intersections), total number of end points (angiogenic tips), and vessels percentage area were analysed using AngioTool software.

PCR and real-time PCR to amplify SMN transcripts

To measure exon 7 skipping in *SMN1* and *SMN2* genes in mouse retinas or HUVECs, total RNA was extracted from cells or tissues using the RNeasy Mini kit (Qiagen). 200 ng RNA sample was used for cDNA synthesis using the high-capacity cDNA synthesis kit (Applied Biosystem). Primers (sequences listed in Supplemental Table 3) were used to amplify full-length (505 bp) and Δ7-SMN (451 bp) transcripts from cDNA. The products were amplified by 30 PCR cycles (94°C for 30 s, 55°C for 30 s and 72°C for 30 s). All PCR products were checked by running on 1% agarose gels.

Quantitative reverse transcription PCR was conducted with the SYBR Green qPCR kit (Eurogentec). Samples were incubated in a 20 µl reaction mix according to manufacturer's instructions. Primers (sequences listed in Supplemental Table 3) were designed to specifically amplify the full-length *SMN* transcripts and Δ7-SMN transcripts. *GAPHD* was used as the

reference gene. Quantitative PCR was performed using StepOne Real Time PCR System (Applied Biosystem). The cycle at which the amount of fluorescence was above the threshold (Ct) was detected. The $2^{-\Delta\Delta Ct}$ method was used for the relative expression analysis.

Western blotting

To measure human SMN protein restored from the human *SMN2* transgene in SMA mice after PMO25 antisense treatment, mouse retinas were isolated from saline treated and PMO25 treated SMA mice where there was no endogenous mouse Smn protein present. Retina tissue was lysed in RIPA lysis buffer (Thermo Fisher Scientific) supplemented with protease inhibitor cocktail (Roche Diagnostics). Protein concentration was measured by bicinchoninic acid (BCA) kit (Thermo Fisher Scientific). Ten micrograms of total protein were loaded into 10% NuPAGE Bis-Tris precast gels (Thermo Fisher Scientific). SMN protein was probed by a mouse anti-SMN monoclonal antibody (610647, BD Biosciences, 1:1000). Mouse Gapdh was used as an internal control and was detected with rabbit anti-GAPDH polyclonal antibody (G9545, Sigma-Aldrich, 1:3000). Membrane was probed with IRDye 800CW goat anti-mouse IgG (926-32210, Li-Cor, 1:15,000) and IRDye 680RD goat anti-rabbit (926-68071, Li-Cor, 1:15,000) secondary antibodies. Blots were developed and analysed with Odyssey Imaging System (Li-Cor).
